# Tristetraprolin Mediates Anti-Inflammatory Effect of Carbon Monoxide against DSS-Induced Colitis

**DOI:** 10.1371/journal.pone.0088776

**Published:** 2014-02-19

**Authors:** Yeonsoo Joe, Md. Jamal Uddin, Min Zheng, Hyo Jeong Kim, Yingqing Chen, Nal Ae Yoon, Gyeong Jae Cho, Jeong Woo Park, Hun Taeg Chung

**Affiliations:** 1 Department of Biological Sciences, University of Ulsan, Ulsan, Korea; 2 Department of Thoracic and Cardiovascular Surgery, Affiliated Hospital of Yanbian University, Yanji, China; 3 Department of Anatomy, School of Medicine and Institute of Health Sciences, Gyeongsang National University, Jinju, Korea; Institut Pasteur de Lille, France

## Abstract

Endogenous carbon monoxide (CO) exerts anti-inflammatory effects. Tristetraprolin (TTP) is known to destabilize pro-inflammatory transcripts. Here we found that exogenous CO enhanced the decay of *TNF-α* mRNA and suppressed TNF-α expression in LPS-activated macrophages from wild-type (WT) mice. However, *TTP* deficiency abrogated the effects of exogenous CO. While CO treatment prior to DSS administration in WT mice significantly reduced inflammatory cytokine levels and colitis, it failed to reduce the pro-inflammatory cytokine levels and colitis in TTP knockout (KO) mice. Our results demonstrate that TTP is a key factor mediating the anti-inflammatory action of CO in DSS-induced colitis.

## Introduction

Endogenous CO is one of the three products of heme degradation created by heme oxygenase-1 (HO-1), the other two being Fe^2+^ and biliverdin [1]. These byproducts have recently been shown to have strong cytoprotective effects. This is thought to result from their anti-inflammatory, anti-apoptotic, and anti-oxidant actions [2]. Interestingly, recent studies have shown that the exogenous application of CO or CO-releasing molecules (CO-RMs) can also confer protective effects in models of inflammatory stress or tissue injury [3]. In intestinal inflammation, exogenous CO reduces the severity of disease activity [4], [5]. Recently, CO-RMs have been used in biological systems to deliver CO in a controlled manner, while keeping carbon monoxy hemoglobin levels stable [6]. CO-RM-derived CO has been shown to inhibit various inflammatory states [7]. In particular, CO-RM ameliorates DSS-induced colitis by inhibition of neutrophil infiltration and TNF-α production [8]. Hence, CO might be a novel and important molecule in the treatment of intestinal inflammation.

Inflammatory bowel diseases (IBD) are inflammatory disorders related to a dysfunction in the innate responses to bacterial products. Multiple genetic defects have been linked to immune dysfunction [9], but the precise pathogenesis of IBD remains unclear. Various models of experimental IBD have been developed to investigate the pathogenesis. The administration of dextran sulfate sodium (DSS) induces a reversible form of colitis in mice [10]. This model is characterized by acute tissue inflammation in the colon and mimics the pathology of human ulcerative colitis. In this colitis model, expression of pro-inflammatory cytokines is upregulated [11]. Since immuno-compromised mice lacking the T and B cell compartments remain susceptible in this model [12], macrophages and dendritic cells (DCs) are proposed to play a central role in the pathogenesis of DSS-induced colitis. Macrophages and intestinal DCs are the first antigen presenting cells (APCs) to sense and respond to exogenous antigens or tissue injury [13] and to activate T cells and induce T cell proliferation in DSS-induced colitis [14].

The inflammatory response has been reported to be modulated by post-transcriptional control [15]. The post-transcriptional control of inflammatory transcripts is strongly dependent on AU-rich element (ARE)-mediated mechanisms [16]. The destabilizing function of AREs is believed to be regulated by ARE-binding proteins [17]. Tristetraprolin (TTP) is an ARE-binding protein that promotes degradation of a number of inflammatory mediators [18]–[20]. TTP-knockout mice develop severe inflammatory arthritis, autoimmune dysfunction, and myeloid hyperplasia, demonstrating the importance of TTP in limiting the inflammatory response [21].

Here we describe a so far unanticipated role of TTP in the anti-inflammatory function of CO in DSS-induced colitis. We found that TTP deficiency exacerbates disease severity and blocks the anti-inflammatory activity of CO in DSS-induced colitis. We provide evidence that CO induces the expression of TTP, and in turn, TTP plays an important role in the CO-induced anti-inflammatory effect through destabilization of *TNF-α* transcripts.

## Methods

### Cells

Peripheral macrophages were isolated from wild-type (WT) and TTP knockout (KO) mice according to previously described methods [22]. Peritoneal macrophages and murine monocyte macrophage cells (RAW264.7) were cultured in DMEM supplemented with 10% FBS. Cells were exposed for 4 h to LPS (1 µg/ml, L-2654, Sigma, USA) in the presence or absence of tricarbonyldichlororuthenium (II) dimer (CORM-2; Sigma, USA) (5, 10, 20, and 50 µM), and TTP and TNF-α levels were determined at the end of the incubation. Inactive CORM-2 (iCORM-2) was used as a negative control [23] to assess whether the effects observed were due to the CO liberated by the CORM-2 or were caused by other components of the molecules.

### Mice and Ethics Statement

TTP knockout (KO) mice were kindly provided by Dr. Perry J Blackshear (Laboratory of Signal Transduction, National Institute of Environmental Health Sciences, USA). Mice were bred in the animal facility at the University of Ulsan and were born and housed in the same room under specific pathogen-free conditions. In all experiments, sex- and age-matched littermates were used as controls. All mice were handled in accordance with the guidelines of the Institutional Animal Care and Use Committee (IACUC) of the Immunomodulation Research Center (IRC), University of Ulsan. All animal procedures were approved by IACUC of IRC (Permit Number: UOU-2012-005). All surgery was performed under sodium pentobarbital anesthesia, and all efforts were made to minimize suffering.

### Dextran Sulfate Sodium (DSS)-mediated Colitis

Acute colitis was induced with 2% (w/v) dextran sulfate sodium (DSS) (36–50 kDa, MP Biomedicals) dissolved in drinking water for 7 days followed by normal drinking water until the end of the experiment. The DSS solutions were made fresh every day.

### CO Inhalation Treatment

After treatment with DSS for 7 days, mice were exposed to CO at a concentration of 250 ppm in an exposure chamber (LB Science, Daejeon, Korea) at room temperature for 3 h a day for 5 days. A CO analyzer (Tongoy Control Technology, Beijing, China) was used to continuously measure the CO levels in the cage in order to maintain the CO concentration at 250 ppm. The animals were divided into three groups: a sham group, a DSS-induced colitis group, and a DSS-induced colitis group treated with CO inhalation.

### Evaluation of Colitis Severity

The clinical activity score was determined daily by scoring changes in animal weight, occult blood positivity, gross bleeding, and stool consistency. We used five grades of weight loss (0, no loss or weight gain; 1, 1–5% loss; 2, 5–10% loss; 3, 10–20% loss; 4, >20% loss), three grades of stool consistency (0, normal; 2, loose; and 4, diarrhea), and three grades of occult blood (0, negative; 2, occult blood-positive; and 4, gross bleeding). These scores were added to generate a clinical activity score ranging from 0 to 12.

After determining the clinical activity score, mice were sacrificed, the entire colon was removed from the cecum to the anus, and the colon length was measured as an indirect marker of inflammation. The distal colon was fixed in 10% buffered formalin for histological analysis. Sections 5 µm thick were prepared and stained with hematoxylin and eosin (H&E).

### Determination of TNF-α Levels

The concentration of TNF-α in the supernatant of cultured cells or mucosal homogenates was analyzed using DuoSet ELISA Development Systems (R&D Systems, Minneapolis, MN, USA).

### Plasmids, Transfections and Luciferase Assay

The genomic sequence of the mouse *TTP* gene (GenBank accession number NT_187034) was used to engineer PCR cloning primers: 5′-GAGCTCTTCTATCTTTCTGTAACCCAC-3′, 5′-CTCGAGTGGCAGAGAGATCCATGGTGG-3′. Underlined letters are restriction enzyme sites. A 1309-base pair (bp) genomic fragment containing the 5′-flanking region of the *TTP* gene was isolated by PCR amplification from mouse genomic DNA. Construct pGL3/mTTP p-1309 contains the -1309 bp promoter region of the mouse TTP gene up to nucleotide +48 base pair (*i.e.,* downstream from the mouse TTP mRNA cap site) inserted into the *Sac*I and *Xho*I sites of the pGL3 basic vector (Promega). A pRL-SV40 *Renilla* luciferase construct was purchased from Promega (E2231).

RAW264.7 cells were transfected with luciferase reporter constructs using the Neon™ transfection system (Invitrogen). Lysates of the transfected cells were mixed with luciferase assay reagent (Promega), and the chemiluminescent signal was measured in a Wallac Victor 1420 Multilabel Counter (EG&G Wallac, Turku, Finland). Firefly luciferase of pGL3/mTTP p-1309 was normalized to *Renilla* luciferase of pRL-SV40 in each sample.

### Actinomycin D-based RNA Kinetic Analysis

Peritoneal macrophages were exposed to 10 µM CORM-2 (Sigma) and 1 µg/ml LPS (Sigma) for 20 min. After incubation for 24 h, cells were incubated with 5 µg/ml actinomycin D (Sigma) to stop transcription, and cells were collected at 0, 15, 30, 45, and 60 min after addition of actinomycin D and analyzed for *TNF-α* mRNA by quantitative RT-PCR.

### Quantitative Real-time PCR and Semi-qRT-PCR

Total RNAs were extracted from cells and colon of WT and TTP KO mice using the RNeasy Mini Kit (Qiagen). For RNA kinetic analysis, 3 µg of DNase I-treated total RNA was reverse transcribed using oligo-dT and Superscript II reverse transcriptase (Invitrogen) according to the manufacturer’s instructions. qRT-PCR was performed by monitoring the increase in fluorescence in real-time of the SYBR Green dye (QIAGEN, Hilden, Germany) using StepOnePlus™ Real-time PCR systems (Applied Biosystems). Semi-qRT-PCR was performed using Taq polymerase (Solgent, Daejeon, Korea) and PCR primer pairs:

GAPDH, 5′-ATGACAACTTTGGCATTGTG-3′ and 5′-CATACTTGGCAGGTTTCTCC-3′; 18S rRNA, 5′-CAGTGAAACTGCGAATGGCT-3′ and 5′-TGCCTTCCTTGGATGTGGTA-3′; KC (CXCL1), 5′-TTGTGCGAAAAGAAGTGCAG-3′ and 5′-TACAAACACAGCCTCCCACA-3′; IL-6, 5′-ACAAGTCGGAGGCTTAATTACACAT-3′ and 5′-TTGCCATTGCACAACTCTTTTC-3′; TNF-α, 5′-AGGCTGCCCCGACTACGT-3′ and 5′-GACTTTCTCCTGGTATGAGATAGCAAA-3′; MIP-2 (CXCL2), 5′-CACTCTCAAGGGCGGTCAAA-3′ and 5′-TACGATCCAGGCTTCCCGGGT-3′.

### Myeloperoxidase (MPO) Assay

The MPO enzyme activity in colonic tissues was analyzed using Mouse Myeloperoxidase DuoSet (R&D Systems, Minneapolis, MN).

### Statistical Analysis

For statistical comparisons, p-values were determined using Student’s t test.

## Results

### CO Induces *TTP* mRNA in Macrophages

**Figure 1:**
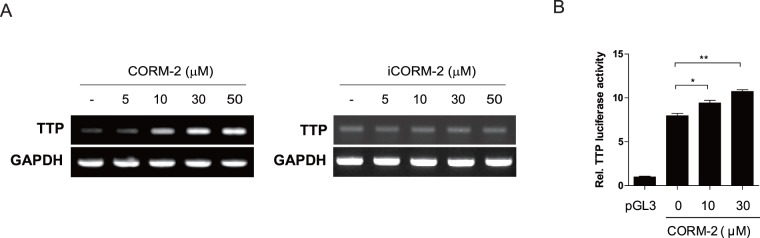
CORM-2 induces TTP promoter activity and *TTP* mRNA in macrophages. A. RAW264.7 cells were incubated with different concentration of CORM-2 or iCORM-2 for 4 h. *TTP* mRNA expression was determined by semi-quantitative RT-PCR. B. RAW264.7 cells were transfected with pGL3/mTTP P-1309 containing the mouse *TTP* promoter. After 24 h, cells were treated with different concentrations of CORM-2 for 4 h, and luciferase activity was determined. The level of firefly luciferase activity was normalized using *Renilla* luciferase activity. The relative luciferase activity is presented as a fold increase over untreated cells. Values are mean ± SD (n = 3). *, *p*<0.05; **, *p<*0.01.

CO has been reported to suppress the production of inflammatory cytokines [7]. However, the molecular basis by which CO mediates such anti-inflammatory functions is unknown. TTP enhances degradation of various inflammatory cytokines [18]–[20]. This prompted us to evaluate the functional link between CO and TTP in macrophages, with the hypothesis that CO increases the expression of TTP, which in turn mediates the anti-inflammatory activity of CO by enhancing the degradation of mRNAs of inflammatory cytokine genes. To test this hypothesis, we initially determined the effect of CORM-2 on the expression level of TTP in macrophages. CORM-2 treatment significantly increased TTP transcripts in a dose-dependent manner (Fig. 1A). This effect appeared to be strictly dependent on the CO released by CORM-2, since iCORM-2, which does not liberate CO, did not have any effect on TTP level (Fig. 1A).

The experiments above indicate that CORM-2 is able to induce *TTP* transcription. We next tested whether CORM-2 was able to transactivate the *TTP* promoter in a reporter assay. RAW264.7 cells were transiently transfected with pGL3/mTTP p-1309 construct containing the −1261 ∼ +48 promoter region of the mouse TTP gene followed by treatment with CORM-2. CORM-2 treatment significantly stimulated the *TTP* promoter at 4 h after the treatment in a dose-dependent manner (Fig. 1B). These data indicate that CORM-2 enhances the *TTP* promoter and thus increases TTP transcript level.

### TTP Deficiency Abrogates the Anti-inflammatory Effect of CORM-2 in Macrophages

**Figure 2:**
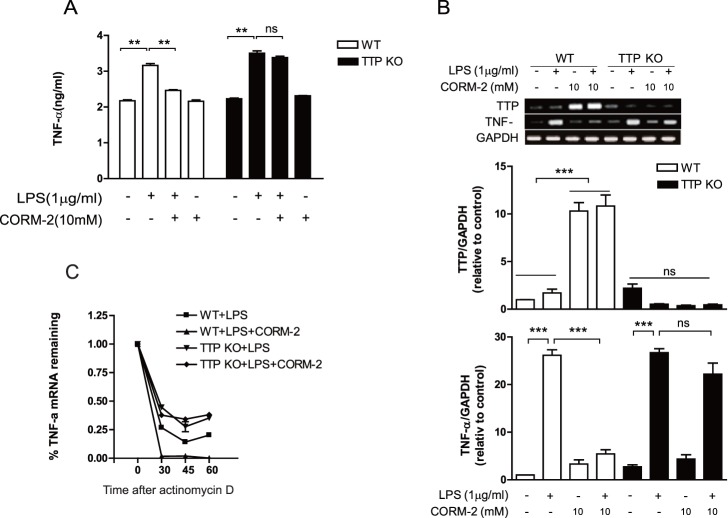
TTP deficiency blocks the anti-inflammatory function of CORM-2 in macrophages. Peritoneal macrophages were harvested from WT and TTP KO mice. Cells were treated with 1 µg/ml LPS in the presence of 10 µM CORM-2 for 4 h. A. TNF-α in the supernatant of cells was determined by ELISA. Data shown are mean ± SD (n = 3). **, *p<*0.01. B. The expression levels of *TTP* and *TNF-α* mRNA were determined by semi-quantitative RT-PCR. C. Expression of *TNF* mRNA in macrophages was determined by quantitative real-time PCR at indicated times after the addition of 5 µg/ml actinomycin D. Values are mean ± SD (n = 3). ***, *p<*0.001. The band densities in the agarose gel were quantified by PhosphorImager, normalized to the internal control GAPDH and expressed as percentage (%) of the value of untreated control cells. Data shown are mean ± SD (n = 3). ***, p<0.005. ns, not significant.

Our next goal was to determine whether TTP deficiency affects the anti-inflammatory effect of CORM-2 in macrophages. To test this, we treated peritoneal macrophages from wild-type and TTP KO mice with LPS in the presence or absence of CORM-2 and analyzed TNF-α expression by ELISA and RT-PCR. LPS treatment caused a significant increase in TNF-α in macrophages from both wild-type and TTP KO mice (Figs. 2A and 2B). However, although CORM-2 significantly inhibited the production of TNF-α induced by LPS in macrophages from wild-type mice, deficiency of TTP blocked the inhibitory effect of CORM-2 on LPS-induced production of TNF-α (Figs. 2A and 2B). These results suggest that TTP deficiency suppresses the anti-inflammatory effect of CORM-2 in macrophages.

We next determined whether CORM-2 could decrease the stability of *TNF-α* mRNA induced by LPS in macrophages. In macrophages from wild-type mice, CORM-2 treatment significantly decreased the stability of *TNF-α* mRNA (Fig. 2C), indicating that the inhibitory effect of CORM-2 on the TNF-α production in macrophages results from enhancing the decay of *TNF-α* mRNA. To determine whether TTP deficiency affects the CORM-2-induced destabilization of *TNF-α* in macrophages, macrophages from TTP KO mice were treated with LPS in the presence or absence of CORM-2. In contrast to the macrophages from wild-type mice, CORM-2 treatment did not decrease the half-life of *TNF-α* mRNA in macrophages from TTP KO mice (Fig. 2C), suggesting that TTP deficiency blocks the CORM-2-induced destabilization of *TNF-α* mRNA.

### TTP Knockout Increases Susceptibility of Mice to DSS-induced Colitis

**Figure 3:**
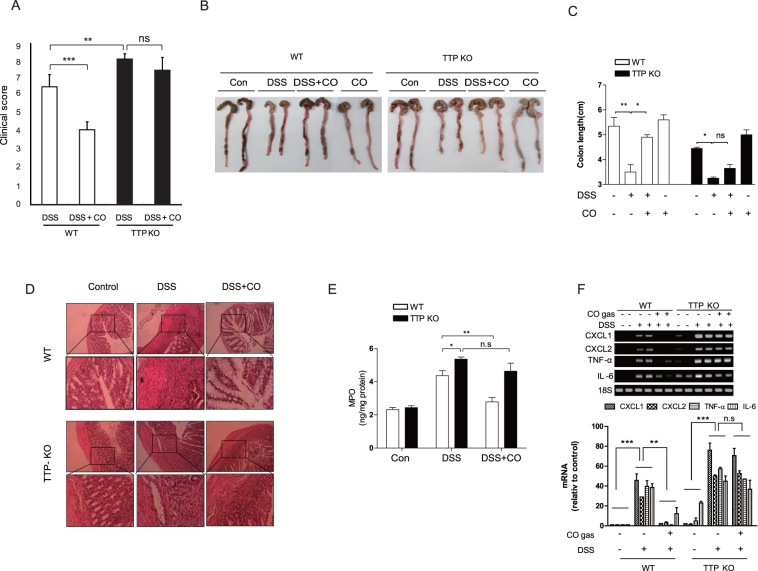
TTP deficiency blocks the anti-inflammatory function of CO in DSS-induced colitis. After treatment with 2% (w/v) DSS for 7 days, WT and TTP KO mice were exposed to CO at a concentration of 250 ppm for 5 days and analyzed for colitis. A. Clinical scores were assessed as described in Materials and Methods. Data are mean ± SD for 5 mice (**, p<0.01; ***, p<0.005). ns, not significant. B and C. Colon length. B. Representative images of 5 tests conducted in each group. C. Data are mean ± SD for 5 mice (*, p<0.05; **, p<0.01). D. Representative H&E sections of each group from colons of WT and TTP KO mice. E. Neutrophil infiltration into the colon, quantified by measuring MPO activity. Data are mean ± SD for 5 mice (*, p<0.05; **, p<0.01). F. Colonic cytokine/chemokine mRNA levels, analyzed by semi-quantitative RT-PCR. Data represent 1 of 3 independent experiments with similar results. The band densities in the agarose gel were quantified by PhosphorImager, normalized to the internal control GAPDH and expressed as percentage (%) of the value of untreated control cells. Data shown are mean ± SD (n = 3). **, p<0.01; ***, p<0.005. ns, not significant.

Administration of DSS increases the levels of pro-inflammatory cytokines [11]. Thus, it is possible to predict that TTP deficiency exacerbate DSS-induced colitis. To test this hypothesis, we assessed the effect of TTP knockout on intestinal inflammation in a DSS-induced colitis model. During the course of DSS treatment, TTP KO mice displayed a higher clinical score determined at day 12 after treatment (Fig. 3A). Colon shortening, a macroscopic parameter of colitis severity, was more pronounced in DSS-treated TTP KO mice than in DSS-treated WT mice (colon length, 3.50±0.13 cm vs. 4.07±0.13 cm; Figs. 3B and 3C). No significant difference was observed in the colon length between untreated WT and TTP KO mice. In addition, histological study showed that DSS treatment resulted in greater loss of epithelial crypts and massive infiltration of inflammatory cells into the mucosa in TTP KO mice than in WT mice (Fig. 3D). The activity of myeloperoxidase (MPO), a marker for neutrophil infiltration, in the colon of DSS-treated TTP KO mice was also increased compared with that in DSS-treated WT mice (Fig. 3E). Next, we determined the effects of TTP deficiency on the DSS-induced production of inflammatory cytokines. It has been reported that DSS can develop colitis in the absence of TNF-α [24]. Thus, we analyzed the levels of pro-inflammatory cytokines including IL-6, CXCL1, and CXCL2 as well as TNF-α. As shown in Fig. 3F, TTP KO mice showed a marked increase in DSS-induced production of TNF-α, IL-6, CXCL1, and CXCL2 in colon tissues. Overall, these results demonstrate that TTP deficiency aggravates inflammation in a mouse model of colitis.

### TTP is Required for the CO-mediated Protection Against DSS-induced Colitis

Based on our findings that CORM-2 increases TTP levels, and in turn, TTP mediates the anti-inflammatory effect of CORM-2 in macrophages and TTP deficiency exacerbates DSS-induced colitis, we hypothesized that exogenous CO and CO-RM may protect mice from DSS-induced colitis through a TTP–dependent mechanism. To assess whether TTP is required for the anti-inflammatory effects of exogenous CO in DSS-induced colitis, we administered 2% DSS to wild-type mice and TTP KO mice with or without exposure to exogenous CO. CO inhalation treatment significantly decreased the levels of inflammatory cytokines (TNF-α and IL-6) and chemokines (CXCL1 and CXCL2) induced by DSS in wild-type mice (Fig. 3F). However, in TTP KO mice, the inhibitory effect of CO on the production of inflammatory cytokines and chemokines was blocked (Fig. 3F). In addition, CO decreased the susceptibility of wild-type mice to DSS-induced colitis as determined by clinical score (Fig. 3A), colon length (Figs. 3B and 3C), and neutrophil infiltration (Figs. 3D and 3E). However, in TTP KO mice, CO treatment did not change the severity of DSS-induced colitis in TTP KO mice (Figs. 3A–F). Collectively, our data suggest that TTP is required for the anti-inflammatory effect of CO in the DSS-induced colitis model.

## Discussion

The administration of CO or CO-RMs can inhibit the production of inflammatory cytokines and protect animals against inflammatory diseases [3]. However, the basic mechanisms underlying the anti-inflammatory function of CO are not clear. In this study, we provide evidence that CO exerts its inhibitory effects through induction of TTP expression in macrophages; CORM-2 increases the *TTP* promoter activity and the expression of TTP, decreases the stability of *TNF-α* mRNA induced by LPS in a TTP-dependent manner, and CO decreases the levels of *TNF-α*, *IL-6*, *CXCL1*, and *CXCL2* induced by LPS, while TTP deficiency blocks the anti-inflammatory effect of CO. These results indicate that TTP induced by CO-RM mediates the anti-inflammatory function of CO-RM in macrophages. Here we did not analyze the mechanisms for the CO-RM-induced expression of TTP. It has been reported that CO activates STAT3 [25] and STAT3 binds to TTP promoter region and induces TTP expression in macrophages [26]. Thus, it is possible to predict that the CO/STAT3 pathway enhances the *TTP* promoter activity and increases *TTP* expression. Further studies are required to elucidate the mechanisms involved in the induction of *TTP* by CO-RM.

The expression of inflammatory cytokines is associated with the development of intestinal inflammation in both animal models of colitis [27] and human IBD [28]. In the DSS-induced colitis model, expression of pro-inflammatory cytokines and chemokines is upregulated [11]. The mRNAs of these inflammatory cytokines contain AREs within their 3′UTR, and their expression can be down-regulated by TTP [18]–[20]. Thus, mice lacking the *TTP* gene may lose their ability to down-regulate the production of inflammatory cytokines in response to tissue damage in the colon, resulting in exacerbated colitis. Consistently, we found that the TTP KO mice treated with DSS displayed increased susceptibility to colitis, decreased colon length, and increased neutrophil infiltration and expression of pro-inflammatory cytokines such as *TNF-α*, *IL-6*, *CXCL1*, and *CXCL2* in the large intestinal mucosa. Considering the facts that these inflammatory cytokines are described for both animal models of colitis [27] and human IBD [28], it is possible to predict that decreased level and/or activity of TTP in ulcerative colitis patients may lead to a defect in resolution of intestinal inflammation and exacerbated IBD symptoms.

It has been reported that CO-RM can protect against DSS-induced colitis [8]. The exacerbated colitis in TTP KO mice led us to test whether CO-RM can ameliorate DSS-induced colitis in TTP KO mice as well. Consistent with a previous report [8], we observed that, in wild-type mice, CO ameliorated DSS-induced colitis by inhibition of neutrophil infiltration and inflammatory cytokine production. However, under conditions of CO exposure, TTP deficiency prevented the anti-inflammatory effect of CO in DSS-induced colitis. Previously it has been reported that HO-1 mediates the inhibitory effect of CO on chronic colitis [4]. Our data indicate that TTP is a key regulator of intestinal inflammatory diseases and CO-mediated anti-inflammatory function. Previously, it has been reported that TTP-knockout mice develop severe inflammatory arthritis, demonstrating the importance of TTP in limiting the inflammatory response [21].

In conclusion, our study with an intestinal inflammation models shows that CO-mediated protection operates in part by inducing TTP, which leads to the down-regulation of inflammatory cytokines and chemokines. TTP deficiency exacerbates DSS-induced colitis and abrogates the protective effect of CO against DSS-induced colitis. Taken together, these data demonstrate for the first time a dependence on TTP in the CO-induced protection against DSS-induced colitis. The finding that TTP plays important roles in protection against DSS-induced colitis makes CO-TTP pathway an attractive candidate for the treatment of ulcerative colitis.

## References

[1] Maines MD (1997) The heme oxygenase system: a regulator of second messenger gases. Annu Rev Pharmacol Toxicol 37: 517–554. 9131263 10.1146/annurev.pharmtox.37.1.517

[2] Morse D, Choi AM (2005) Heme oxygenase-1: from bench to bedside. Am J Respir Crit Care Med 172: 660–670. 15901614 10.1164/rccm.200404-465SO

[3] Motterlini R, Otterbein LE (2010) The therapeutic potential of carbon monoxide. Nat Rev Drug Discov 9: 728–743. 20811383 10.1038/nrd3228

[4] Hegazi RA, Rao KN, Mayle A, Sepulveda AR, Otterbein LE, et al (2005) Carbon monoxide ameliorates chronic murine colitis through a heme oxygenase 1-dependent pathway. J Exp Med 202: 1703–1713. 16365149 10.1084/jem.20051047 PMC2212966

[5] Naito Y, Uchiyama K, Takagi T, Yoshikawa T (2012) Therapeutic potential of carbon monoxide (CO) for intestinal inflammation. Curr Med Chem 19: 70–76. 22300078 10.2174/092986712803413935

[6] Motterlini R, Clark JE, Foresti R, Sarathchandra P, Mann BE, et al (2002) Carbon monoxide-releasing molecules: characterization of biochemical and vascular activities. Circ Res 90: E17–24. 11834719 10.1161/hh0202.104530

[7] Cepinskas G, Katada K, Bihari A, Potter RF (2008) Carbon monoxide liberated from carbon monoxide-releasing molecule CORM-2 attenuates inflammation in the liver of septic mice. Am J Physiol Gastrointest Liver Physiol 294: G184–191. 17991708 10.1152/ajpgi.00348.2007

[8] Takagi T, Naito Y, Uchiyama K, Suzuki T, Hirata I, et al (2011) Carbon monoxide liberated from carbon monoxide-releasing molecule exerts an anti-inflammatory effect on dextran sulfate sodium-induced colitis in mice. Dig Dis Sci 56: 1663–1671. 21086163 10.1007/s10620-010-1484-y

[9] Xavier RJ, Podolsky DK (2007) Unravelling the pathogenesis of inflammatory bowel disease. Nature 448: 427–434. 17653185 10.1038/nature06005

[10] Okayasu I, Hatakeyama S, Yamada M, Ohkusa T, Inagaki Y, et al (1990) A novel method in the induction of reliable experimental acute and chronic ulcerative colitis in mice. Gastroenterology 98: 694–702. 1688816 10.1016/0016-5085(90)90290-h

[11] Yan Y, Kolachala V, Dalmasso G, Nguyen H, Laroui H, et al (2009) Temporal and spatial analysis of clinical and molecular parameters in dextran sodium sulfate induced colitis. PLoS One 4: e6073. 19562033 10.1371/journal.pone.0006073 PMC2698136

[12] Dieleman LA, Ridwan BU, Tennyson GS, Beagley KW, Bucy RP, et al (1994) Dextran sulfate sodium-induced colitis occurs in severe combined immunodeficient mice. Gastroenterology 107: 1643–1652. 7958674 10.1016/0016-5085(94)90803-6

[13] Kelsall B (2008) Recent progress in understanding the phenotype and function of intestinal dendritic cells and macrophages. Mucosal Immunol 1: 460–469. 19079213 10.1038/mi.2008.61 PMC4780321

[14] Shintani N, Nakajima T, Sugiura M, Murakami K, Nakamura N, et al (1997) Proliferative effect of dextran sulfate sodium (DSS)-pulsed macrophages on T cells from mice with DSS-induced colitis and inhibition of effect by IgG. Scand J Immunol 46: 581–586. 9420621 10.1046/j.1365-3083.1997.d01-169.x

[15] Stoecklin G, Anderson P (2006) Posttranscriptional mechanisms regulating the inflammatory response. Adv Immunol 89: 1–37. 16682271 10.1016/S0065-2776(05)89001-7

[16] Khabar KS (2005) The AU-rich transcriptome: more than interferons and cytokines, and its role in disease. J Interferon Cytokine Res 25: 1–10. 15684617 10.1089/jir.2005.25.1

[17] Shyu AB, Wilkinson MF (2000) The double lives of shuttling mRNA binding proteins. Cell 102: 135–138. 10943833 10.1016/s0092-8674(00)00018-0

[18] Carballo E, Lai WS, Blackshear PJ (1998) Feedback inhibition of macrophage tumor necrosis factor-alpha production by tristetraprolin. Science 281: 1001–1005. 9703499 10.1126/science.281.5379.1001

[19] Kratochvill F, Machacek C, Vogl C, Ebner F, Sedlyarov V, et al (2011) Tristetraprolin-driven regulatory circuit controls quality and timing of mRNA decay in inflammation. Mol Syst Biol 7: 560. 22186734 10.1038/msb.2011.93 PMC3737733

[20] Molle C, Zhang T, Ysebrant de Lendonck L, Gueydan C, Andrianne M, et al (2013) Tristetraprolin regulation of interleukin 23 mRNA stability prevents a spontaneous inflammatory disease. J Exp Med 210: 1675–1684. 23940256 10.1084/jem.20120707 PMC3754859

[21] Taylor GA, Carballo E, Lee DM, Lai WS, Thompson MJ, et al (1996) A pathogenetic role for TNF alpha in the syndrome of cachexia, arthritis, and autoimmunity resulting from tristetraprolin (TTP) deficiency. Immunity 4: 445–454. 8630730 10.1016/s1074-7613(00)80411-2

[22] Kumagai K, Itoh K, Hinuma S, Tada M (1979) Pretreatment of plastic Petri dishes with fetal calf serum. A simple method for macrophage isolation. J Immunol Methods 29: 17–25. 114591 10.1016/0022-1759(79)90121-2

[23] Sawle P, Foresti R, Mann BE, Johnson TR, Green CJ, et al (2005) Carbon monoxide-releasing molecules (CO-RMs) attenuate the inflammatory response elicited by lipopolysaccharide in RAW264.7 murine macrophages. Br J Pharmacol 145: 800–810. 15880142 10.1038/sj.bjp.0706241 PMC1576195

[24] Naito Y, Takagi T, Handa O, Ishikawa T, Nakagawa S, et al (2003) Enhanced intestinal inflammation induced by dextran sulfate sodium in tumor necrosis factor-alpha deficient mice. J Gastroenterol Hepatol 18: 560–569. 12702049 10.1046/j.1440-1746.2003.03034.x

[25] Zhang X, Shan P, Alam J, Fu XY, Lee PJ (2005) Carbon monoxide differentially modulates STAT1 and STAT3 and inhibits apoptosis via a phosphatidylinositol 3-kinase/Akt and p38 kinase-dependent STAT3 pathway during anoxia-reoxygenation injury. J Biol Chem 280: 8714–8721. 15590660 10.1074/jbc.M408092200

[26] Joe Y, Kim HJ, Kim S, Chung J, Ko MS, et al (2011) Tristetraprolin mediates anti-inflammatory effects of nicotine in lipopolysaccharide-stimulated macrophages. J Biol Chem 286: 24735–24742. 21606497 10.1074/jbc.M110.204859 PMC3137049

[27] Garside P (1999) Cytokines in experimental colitis. Clin Exp Immunol 118: 337–339. 10594548 10.1046/j.1365-2249.1999.01088.x PMC1905439

[28] Autschbach F, Giese T, Gassler N, Sido B, Heuschen G, et al (2002) Cytokine/chemokine messenger-RNA expression profiles in ulcerative colitis and Crohn’s disease. Virchows Arch 441: 500–513. 12447682 10.1007/s00428-002-0684-z

